# Crystal structure and Hirshfeld surface analysis of di­chlorido­[2-(3-cyclo­pentyl-1,2,4-triazol-5-yl-κ*N*^4^)pyridine-κ*N*]palladium(II) di­methyl­formamide monosolvate

**DOI:** 10.1107/S2056989024007801

**Published:** 2024-08-16

**Authors:** Viktoriya V. Dyakonenko, Dmytro M. Khomenko, Roman O. Doroshchuk, Ganna V. Ivanova, Rostyslav D. Lampeka

**Affiliations:** a SSI "Institute for Single Crystals" of National Academy of Sciences of Ukraine, Nauki Ave 60, Kharkiv 61001, Ukraine; b V I Vernadskii Institute of General and Inorganic Chemistry of National, Academy of Sciences of Ukraine, Prospect Palladina 32/34 03680 Kyiv Ukraine; c Department of Chemistry, Taras Shevchenko National University of Kyiv, 12, Hetman Pavlo Skoropadskyi st.,01033 Kyiv, Ukraine; d Enamine Ltd. (www.enamine.net), Winston Churchill str. 78, 02094 Kyiv, Ukraine; Universidade de Sâo Paulo, Brazil

**Keywords:** crystal structure, palladium(II), di­chloro­palladium, 1,2,4-triazole, Hirshfeld surface analysis

## Abstract

This study presents the synthesis, characterization and Hirshfeld surface analysis of the title mononuclear complex, [PdCl_2_(C_12_H_14_N_4_)]·C_3_H_7_NO. The compound crystalizes in the *P*2_1_/*c* space group of the monoclinic system. The asymmetric unit contains one neutral complex Pd(H*L*^c-Pe^)Cl_2_ [H*L*^c-Pe^ is 2-(3-cyclo­pentyl-1,2,4-triazol-5-yl)pyridine] and one mol­ecule of DMF as a solvate. The Pd atom has a square-planar coordination. In the crystal, mol­ecules are linked by inter­molecular N—H⋯O and C—H⋯N hydrogen bonds, forming layers parallel to the *bc* plane. A Hirshfeld surface analysis showed that the H⋯H contacts dominate the crystal packing with a contribution of 41.4%. The contribution of the N⋯H/H⋯N and H⋯O/O⋯H inter­actions is somewhat smaller, amounting to 12.4% and 5%, respectively.

## Abstract 2

The crystal structure of mononuclear di­chlorido­palladium(II) complex with 2-(3-cyclo­pentyl-1,2,4-triazol-5-yl)pyridine is reported and discussed.

## 1. Chemical context

In recent years, square-planar coordination compounds of *d*^8^ metals with N-containing ligands have been widely investigated as effective catalysts and pre-catalysts in organic transformations (Kumbhar, 2017 ▸; Zakharchenko *et al.*, 2019 ▸; Jindabot *et al.*, 2014 ▸; Jiao *et al.*, 2020 ▸), components for optoelectronic devices (Cuerva *et al.*, 2014 ▸, 2018 ▸, 2023 ▸; Cuerva, Campo, Cano & Schmidt, 2019 ▸; Cuerva, Campo, Cano & Lodeiro, 2019 ▸), and analogs of anti­cancer drugs (Abu-Surrah & Kettunen, 2006 ▸; Ouellette *et al.*, 2019 ▸; Jakubowski *et al.*, 2020 ▸; Zakharchenko *et al.*, 2021 ▸; Ohorodnik *et al.*, 2023 ▸). Concurrently, functionalized pyridyl-azole-based ligands have been used in coordination chemistry as chelating polydentate ligands for obtaining various types of metal complexes with potential applications in similar fields. Complexes of di­chloro­palladium with functionalized pyridyl-1,2,3-triazole ligands were shown to be effective pre-catalysts with a broad functional group tolerance for cross-coupling reactions (Jindabot *et al.*, 2014 ▸; Jiao *et al.*, 2020 ▸). A series of metallomesogens of dihalide Pd^II^ and Pt^II^ compounds containing pyridyl-pyrazole ligands have been obtained in the context of the investigation of these complexes as 2D proton-conducting materials under anhydrous conditions (Cuerva *et al.*, 2014 ▸, 2018 ▸; Cuerva, Campo, Cano, & Schmidt, 2019 ▸). In subsequent studies, coordination compounds of this type were used as building blocks (precursors) for the synthesis of metallomesogens with structural asymmetry, which extends the known ranges of mesophases (Cuerva *et al.*, 2018 ▸, 2023 ▸; Cuerva, Campo, Cano, & Lodeiro, 2019 ▸). Furthermore, Pt^II^ metallomesogens exhibit photophysical multi-stimuli-responsive properties (Cuerva, Campo, Cano, & Lodeiro, 2019 ▸). The complexes of *d*^8^ metals with pyridyl-azole-based ligands have been explored in cancer therapy as analogues of cisplatin; their application is limited by the severe side effects and development of drug resistance. The combination of *d*^8^ metals and chelating pyridyl-azole-based ligands should lead to an increase in the stability of the corresponding complexes and to a decrease in hydrolysis in biological media and, as a result, to a decrease in the toxicity of the resulting compounds (Abu-Surrah & Kettunen, 2006 ▸). Previous studies demonstrated that the complexes of Pd and Pt with hydro­phobic pyridyl-azole-based ligands have certain anti­cancer activity against various types of tumour cells *in vitro* (Ouellette *et al.*, 2019 ▸; Jakubowski *et al.*, 2020 ▸; Zakharchenko *et al.*, 2021 ▸; Ohorodnik *et al.*, 2023 ▸). Our previous research of six dichloride Pd^II^ complexes based on 5-substituted 3-(2-pyrid­yl)-5-alkyl-1,2,4-triazoles was reported. The evaluation of ^1^H NMR spectroscopic data was focused on three types of proton signals of ligands and complexes located near the coordination centre and discussed in the context of the influence that cyclo­alkyl substituents have on intra­molecular inter­actions, being also supported by X-ray data for Pd^II^ complexes (Ivanova *et al.*, 2024 ▸). We report herein the crystal structure, including characterization of the inter­molecular contacts by Hirshfeld surface analysis, of a new mononuclear di­chloro­palladium(II) complex with 2-(3-cyclo­pentyl-1,2,4-triazol-5-yl)pyridine.
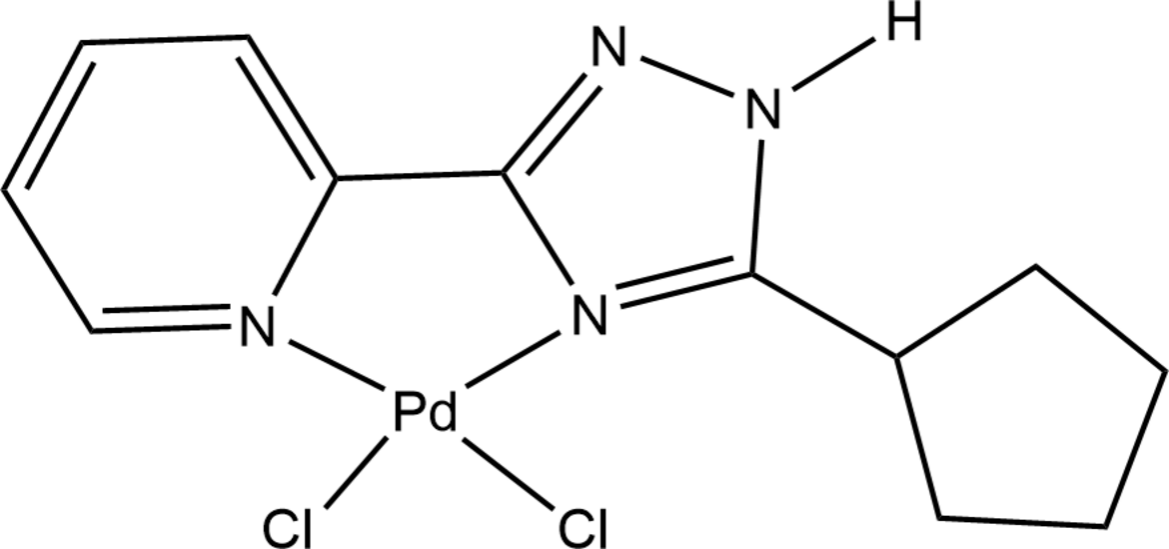


## 2. Structural commentary

**Figure 1:**
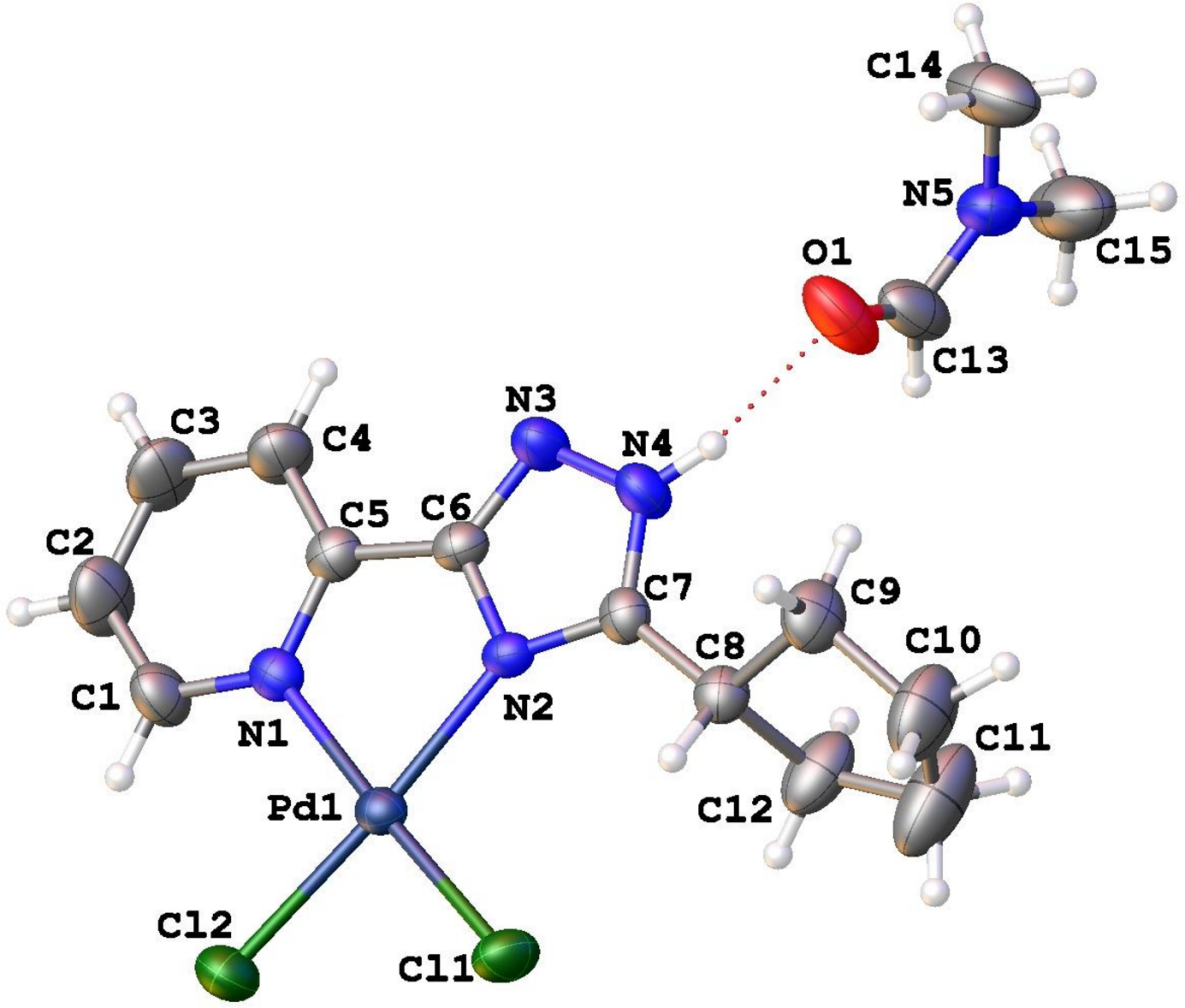
The mol­ecular structure of the title compound, showing the atom labelling and displacement ellipsoids drawn at the 50% probability level.

The title compound crystalizes in the *P*2_1_/*c* space group of the monoclinic system. The asymmetric unit contains one neutral complex Pd(H*L*^c-Pe^)Cl_2_ [H*L*^c-Pe^ is 2-(3-cyclo­pentyl-1,2,4-triazol-5-yl)pyridine] and one mol­ecule of DMF as a solvate. The mol­ecular structure of title compound is shown in Fig. 1 ▸.

The Pd atom has a square-planar environment formed by the bidentate coordination of two nitro­gen atoms of the H*L*^c-Pe^ ligand and two chlorine atoms. The deviation of the Pd atom from the mean-square plane defined by through the Cl1/Cl2/N1/N2 atoms (r.m.s.d. = 0.002 Å) is −0.0164 (11) Å. The Pd—N and Pd—Cl bond distances are 2.038 (3) and 2.061 (3) Å and 2.2811 (11) and 2.2837 (10) Å, respectively (Table 1 ▸). This structure of the title complex is in very good agreement with previously described Pd complexes with a similar coordination (Khomenko *et al.*, 2009 ▸; Zakharchenko *et al.*, 2021 ▸)

The five-membered ring is rotated relative to the plane of the pyridine-triazole fragment and is in the *ac* conformation relative to the C7—N4 bond of the triazole ring [the N4—C7—C8—C12 torsion angle is −91.1 (5)°]. The five-membered ring is in an envelope conformation. Atom C8 deviates by 0.564 (8) Å from the mean square plane through the remaining ring atoms (r.m.s.d. = 0.04Å).

## 3. Supra­molecular features

**Figure 2:**
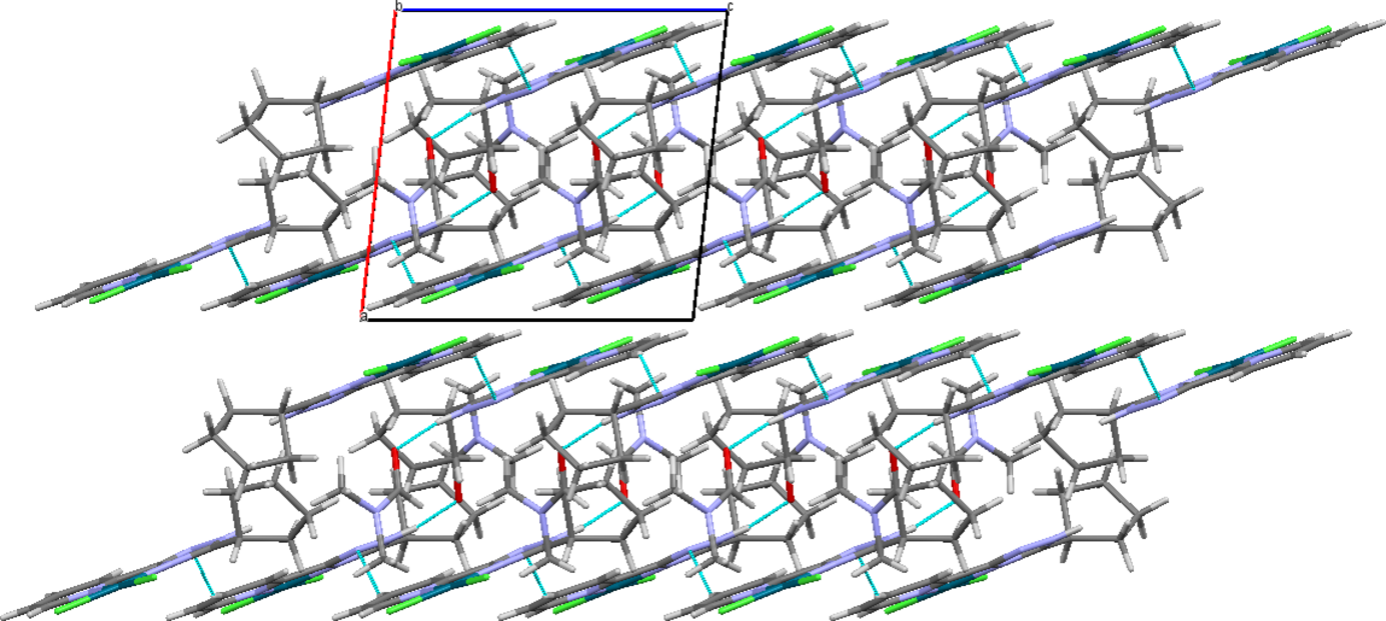
Crystal packing of the title compound.

In the crystal, Pd(H*L*^c-Pe^)Cl_2_ complex mol­ecules, and also mol­ecules of the complex and mol­ecules of DMF are linked by N—H⋯O and C—H⋯N hydrogen bonds (Table 2 ▸), forming layers parallel to the *bc* plane (Fig. 2 ▸).

## 4. Hirshfeld surface analysis and finger print plots

**Figure 3:**
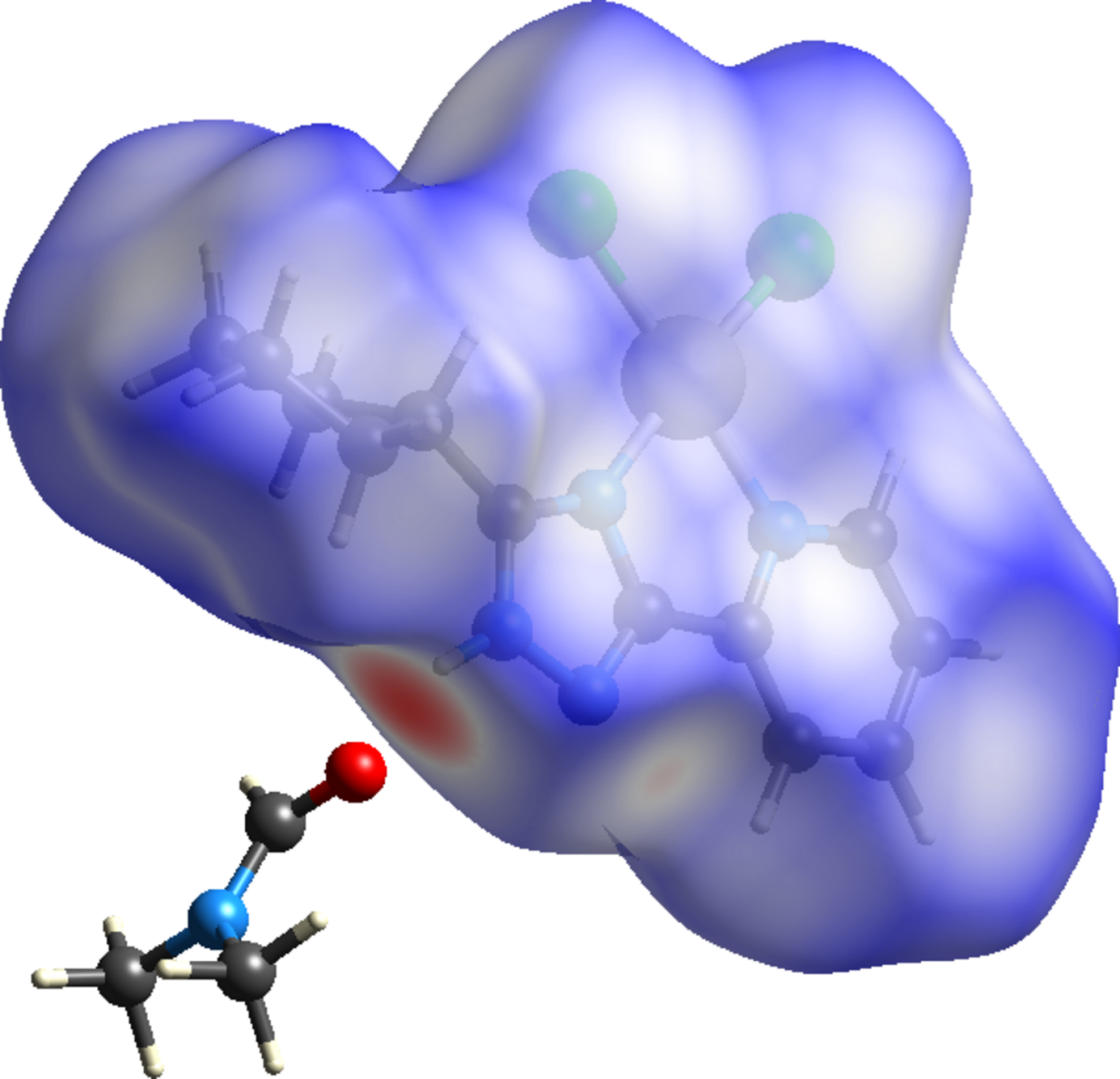
Three-dimensional Hirshfeld surface of title compound mapped over *d*_norm_.

The inter­molecular inter­actions in the crystal structure of the title compound have been analysed by means of the *d*_norm_ property (Fig. 3 ▸) mapped over the Hirshfeld surface (Spackman & Jayatilaka, 2009 ▸), which was calculated using the *CrystalExplorer21* program (Spackman *et al.*, 2021 ▸). The strongest contacts, which are visualized on the Hirshfeld surface as the dark-red spots, correspond to the N—H⋯O hydrogen bond between the complex mol­ecule and the DMF solvent mol­ecule. The lighter red spots correspond to H⋯N/N⋯H inter­actions. The majority of the inter­molecular inter­actions of the title compound are weak, and are represented in blue on the Hirshfeld surface.

**Figure 4:**
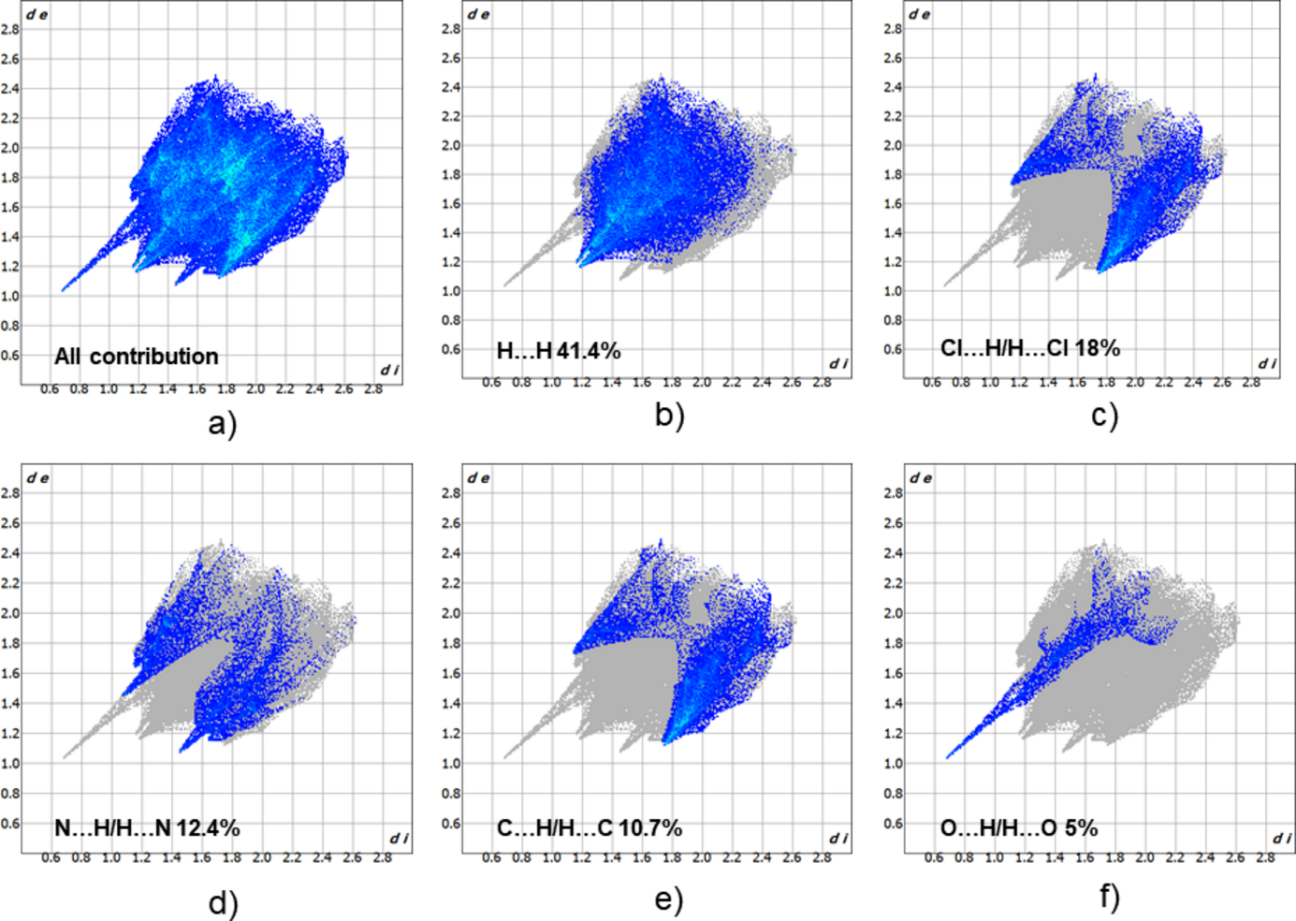
Two-dimensional fingerprint plots for title compound showing (*a*) all inter­actions, and (*b*)–(*f*) delineated into contributions from specific contacts (blue areas) [*d*_e_ and d_i_ represent the distances from a point on the Hirshfeld surface to the nearest atoms outside (external) and inside (inter­nal) the surface, respectively].

For further exploration of the inter­mol­ecular inter­actions, two-dimensional fingerprint plots (McKinnon *et al.*, 2007 ▸) were generated, as shown in Fig. 4 ▸. The H⋯H inter­actions with a contribution of 41.4% have a significant effect on the consolidation in the solid state. The Cl⋯H/H⋯Cl (18.0%), N⋯H/H⋯N (12.4%), C⋯H/H⋯C (10.7%), O⋯H/H⋯O (5%), Cl⋯C/C⋯Cl (4.5%) and N⋯Cl/Cl⋯N (2.5%) inter­actions are less impactful in comparison.

## 5. Database survey

A search of the Cambridge Structural Database (CSD, Version 5.45, updated March 2024; Groom *et al.*, 2016 ▸) found only eleven structures containing the Pd atom coordinated to two Cl atoms and a pyridine-triazole fragment. Of these, seven structures contain a 1,2,3-triazol fragment (Ervithayasuporn *et al.*, 2015 ▸; Ervithayasuporn, 2016 ▸; Schweinfurth *et al.*, 2011 ▸; Yano *et al.*, 2012 ▸; Jindabot *et al.*, 2014 ▸; Schweinfurth *et al.*, 2009 ▸; Lang *et al.*, 2012 ▸) and four structures contain a 1,2,4-triazol fragment (Khomenko *et al.*, 2009 ▸; Zakharchenko *et al.*, 2021 ▸; Ohorodnik *et al.*, 2023 ▸). All of the structures have a square-planar coordination of the Pd atom. The Pd—N and Pd—Cl bond distances vary from 1.999 (2)–2.066 (3) Å and 2.264 (2)–2.293 (2)Å, respectively.

## 6. Synthesis and crystallization

To obtain the complex Pd(H*L*^c-Pe^)Cl_2_·DMF, 0.2 mmol of pre-synthesized Pd(H*L*^c-Pe^)Cl_2_ (Ivanova *et al.*, 2024 ▸) was dissolved in 1 ml of DMF and salted out with 1 ml of MTBE (methyl *tert*-butyl ether) at room temperature for 72 h, affording yellow crystals. The crystals were collected by filtration.

**Figure 5:**
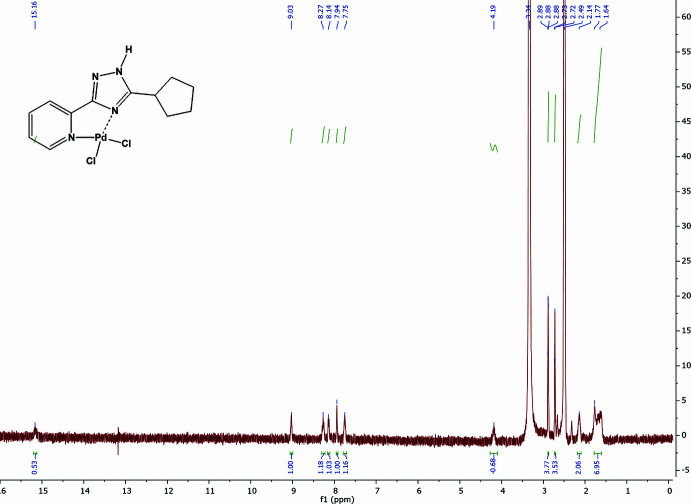
^1^H NMR spectrum of Pd(H*L*^c-Pe^)Cl_2_ in DMSO-*d_6_*.

**Figure 6:**
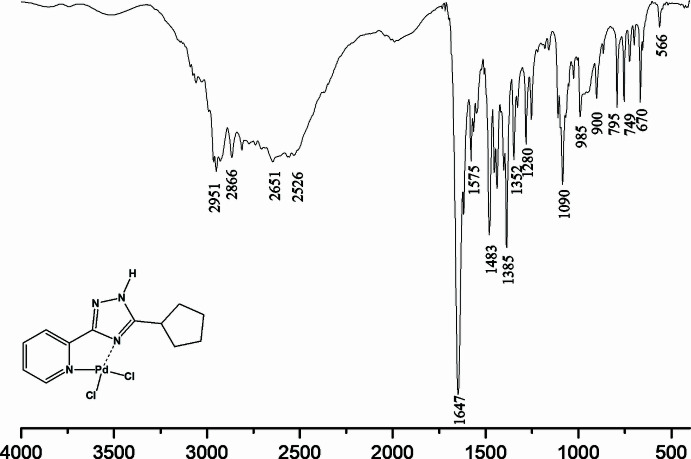
IR spectrum for Pd(H*L*^c*-*Pe^)Cl_2_.

**Pd(H*****L*****^c-Pe^)Cl_2_.** Yield 66%. m. p. >523 K decomp. ^1^H NMR (400 MHz, DMSO-*d_6_*) *δ*: 15.17 (*br s*, 1H, NH), 9.04 (*d*, *J* = 5.6 Hz, 1H, Py-H^6^), 8.28 (*t*, *J* = 7.7 Hz, 1H, Py-H^4^), 8.15 (*d*, *J* = 8.1 Hz, 1H, Py-H^3^), 7.76 (*t*, *J* = 6.0 Hz, 1H, Py-H^5^), 4.20 (*m*, 1H, H^9^), 2.15 (*m*, 2H, H^c-Pe^), 1.78–1.62 (*m*, 6H, H^c-Pe^) ppm (Fig. 5 ▸). IR (KBr, cm^−1^): 3457, 3250, 2945, 2873, 1621, 1543, 1470, 1287, 1090, 788, 723, 467 (Fig. 6 ▸). Elemental analysis: Analysis calculated for C_12_H_14_Cl_2_N_4_Pd (391.58): C, 36.81%; H, 3.60%; N, 14.31%. Found: C: 36.65% H: 3.52% N: 14.43%.

## 7. Refinement

Crystal data, data collection and structure refinement details are summarized in Table 3 ▸. The H atoms were placed in calculated positions and refined using a riding model with *U*_iso_(H) = *nU*_eq_ of the carrier atom (*n* = 1.5 for methyl groups and *n* = 1.2 for other hydrogen atoms). The *U*_ij_ values of the C atoms of the five-membered ring were restrained to be similar to each other (within a standard deviation of 0.02 Å^2^).

## Supplementary Material

Crystal structure: contains datablock(s) I. DOI: 10.1107/S2056989024007801/ex2086sup1.cif

Structure factors: contains datablock(s) I. DOI: 10.1107/S2056989024007801/ex2086Isup2.hkl

CCDC reference: 2376338

Additional supporting information:  crystallographic information; 3D view; checkCIF report

## Figures and Tables

**Table 1:** Selected bond lengths (Å)

Pd1—Cl1	2.2811 (11)	Pd1—N1	2.061 (3)
Pd1—Cl2	2.2837 (10)	Pd1—N2	2.038 (3)

**Table 2:** Hydrogen-bond geometry (Å, °)

*D*—H⋯*A*	*D*—H	H⋯*A*	*D*⋯*A*	*D*—H⋯*A*
N4—H4⋯O1	0.86	1.85	2.679 (4)	161
C3—H3⋯N3^i^	0.93	2.66	3.471 (6)	146

Symmetry code: (i) 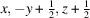.

**Table 3:** Experimental details

Crystal data	
Chemical formula	[PdCl_2_(C_12_H_14_N_4_)]·C_3_H_7_NO
*M*_r_	464.67
Crystal system, space group	Monoclinic, *P*2_1_/*c*
Temperature (K)	296
*a*, *b*, *c* (Å)	9.3964 (8), 20.2572 (16), 9.9822 (7)
β (°)	96.358 (2)
*V* (Å^3^)	1888.4 (3)
*Z*	4
Radiation type	Mo *K*α
μ (mm^−1^)	1.28
Crystal size (mm)	0.4 × 0.2 × 0.15

Data collection	
Diffractometer	Bruker APEXII CCD
Absorption correction	Multi-scan (*SADABS*; Krause *et al.*, 2015 ▸)
*T*_min_, *T*_max_	0.533, 0.746
No. of measured, independent and observed [*I* > 2σ(*I*)] reflections	13789, 4304, 3345
*R*_int_	0.046
(sin θ/λ)_max_ (Å^−1^)	0.650

Refinement	
*R*[*F*^2^ > 2σ(*F*^2^)], *wR*(*F*^2^), *S*	0.051, 0.093, 1.12
No. of reflections	4304
No. of parameters	219
No. of restraints	30
H-atom treatment	H-atom parameters constrained
Δρ_max_, Δρ_min_ (e Å^−3^)	0.64, −0.94

Computer programs: *APEX2* and *SAINT* (Bruker, 2014 ▸), *SHELXT2018/2* (Sheldrick, 2015*a* ▸), *SHELXL2019/3* (Sheldrick, 2015*b* ▸) and *OLEX2* 1.5 (Dolomanov *et al.*, 2009 ▸).

## supplementary crystallographic information

### Dichlorido[2-(3-cyclopentyl-1,2,4-triazol-5-yl-κN4)pyridine-κN]palladium(II) dimethylformamide monosolvate Crystal data

**Table:**

[PdCl_2_(C_12_H_14_N_4_)]·C_3_H_7_NO	*F*(000) = 936
*M**_r_* = 464.67	*D*_x_ = 1.634 Mg m^−^^3^
Monoclinic, *P*2_1_/*c*	Mo *K*α radiation, λ = 0.71073 Å
*a* = 9.3964 (8) Å	Cell parameters from 6028 reflections
*b* = 20.2572 (16) Å	θ = 2.3–30.3°
*c* = 9.9822 (7) Å	µ = 1.28 mm^−^^1^
β = 96.358 (2)°	*T* = 296 K
*V* = 1888.4 (3) Å^3^	Block, orange
*Z* = 4	0.4 × 0.2 × 0.15 mm

### Dichlorido[2-(3-cyclopentyl-1,2,4-triazol-5-yl-κN4)pyridine-κN]palladium(II) dimethylformamide monosolvate Data collection

**Table:**

Bruker APEXII CCD diffractometer	3345 reflections with *I* > 2σ(*I*)
Graphite monochromator	*R*_int_ = 0.046
φ and ω scans	θ_max_ = 27.5°, θ_min_ = 2.0°
Absorption correction: multi-scan (SADABS; Krause *et al.*, 2015)	*h* = −12→11
*T*_min_ = 0.533, *T*_max_ = 0.746	*k* = −23→26
13789 measured reflections	*l* = −10→12
4304 independent reflections	

### Dichlorido[2-(3-cyclopentyl-1,2,4-triazol-5-yl-κN4)pyridine-κN]palladium(II) dimethylformamide monosolvate Refinement

**Table:**

Refinement on *F*^2^	Primary atom site location: dual
Least-squares matrix: full	Hydrogen site location: inferred from neighbouring sites
*R*[*F*^2^ > 2σ(*F*^2^)] = 0.051	H-atom parameters constrained
*wR*(*F*^2^) = 0.093	*w* = 1/[σ^2^(*F*_o_^2^) + (0.0356*P*)^2^ + 0.2719*P*] where *P* = (*F*_o_^2^ + 2*F*_c_^2^)/3
*S* = 1.12	(Δ/σ)_max_ = 0.002
4304 reflections	Δρ_max_ = 0.64 e Å^−^^3^
219 parameters	Δρ_min_ = −0.94 e Å^−^^3^
30 restraints	

### Dichlorido[2-(3-cyclopentyl-1,2,4-triazol-5-yl-κN4)pyridine-κN]palladium(II) dimethylformamide monosolvate Special details

**Table:**

Geometry. All esds (except the esd in the dihedral angle between two l.s. planes) are estimated using the full covariance matrix. The cell esds are taken into account individually in the estimation of esds in distances, angles and torsion angles; correlations between esds in cell parameters are only used when they are defined by crystal symmetry. An approximate (isotropic) treatment of cell esds is used for estimating esds involving l.s. planes.
Refinement. Using Olex2 (Dolomanov *et al.*, 2009), the structure was solved with the SHELXT (Sheldrick, 2018) structure solution program using Intrinsic Phasing and refined with the SHELXL (Sheldrick, 2015) refinement package. Full-matrix least-squares refinement against F^2^ in anisotropic approximation was used for non-hydrogen atoms.

### Dichlorido[2-(3-cyclopentyl-1,2,4-triazol-5-yl-κN4)pyridine-κN]palladium(II) dimethylformamide monosolvate Fractional atomic coordinates and isotropic or equivalent isotropic displacement parameters (Å^2^)

**Table:**

	*x*	*y*	*z*	*U*_iso_*/*U*_eq_	
Pd1	0.14161 (3)	0.51528 (2)	0.63841 (3)	0.02837 (10)	
Cl1	0.17109 (12)	0.61436 (5)	0.53648 (11)	0.0457 (3)	
Cl2	0.06163 (12)	0.56975 (5)	0.81588 (11)	0.0475 (3)	
N1	0.1217 (3)	0.42381 (15)	0.7248 (3)	0.0309 (7)	
N2	0.2130 (3)	0.45857 (15)	0.4909 (3)	0.0261 (7)	
N3	0.2590 (4)	0.35403 (16)	0.4333 (3)	0.0399 (8)	
N4	0.2936 (4)	0.39792 (16)	0.3393 (3)	0.0382 (8)	
H4	0.328939	0.386935	0.266629	0.046*	
C1	0.0774 (5)	0.4111 (2)	0.8460 (4)	0.0450 (11)	
H1	0.049967	0.446010	0.898062	0.054*	
C2	0.0718 (5)	0.3477 (2)	0.8945 (5)	0.0596 (14)	
H2	0.040242	0.339994	0.978152	0.072*	
C3	0.1129 (5)	0.2958 (2)	0.8188 (5)	0.0582 (13)	
H3	0.109421	0.252721	0.850674	0.070*	
C4	0.1593 (5)	0.3083 (2)	0.6954 (4)	0.0469 (11)	
H4A	0.188724	0.273951	0.642997	0.056*	
C5	0.1612 (4)	0.37237 (19)	0.6510 (4)	0.0330 (9)	
C6	0.2103 (4)	0.39271 (19)	0.5233 (4)	0.0312 (9)	
C7	0.2667 (4)	0.4598 (2)	0.3727 (4)	0.0313 (8)	
C8	0.2970 (4)	0.5186 (2)	0.2913 (4)	0.0344 (9)	
H8	0.225634	0.552496	0.305020	0.041*	
C9	0.2950 (5)	0.5072 (2)	0.1405 (4)	0.0501 (11)	
H9A	0.345162	0.466919	0.122290	0.060*	
H9B	0.197656	0.504772	0.096855	0.060*	
C10	0.3716 (6)	0.5671 (3)	0.0933 (5)	0.0771 (16)	
H10A	0.420108	0.556242	0.015282	0.093*	
H10B	0.304162	0.602525	0.069144	0.093*	
C11	0.4769 (6)	0.5871 (3)	0.2084 (6)	0.0959 (19)	
H11A	0.468558	0.633999	0.225607	0.115*	
H11B	0.573529	0.578154	0.187875	0.115*	
C12	0.4455 (5)	0.5479 (3)	0.3309 (5)	0.0609 (13)	
H12A	0.445256	0.576371	0.409077	0.073*	
H12B	0.515995	0.513355	0.351091	0.073*	
O1	0.4202 (4)	0.33801 (18)	0.1446 (3)	0.0690 (10)	
N5	0.6183 (4)	0.28119 (17)	0.1094 (4)	0.0473 (9)	
C13	0.5478 (6)	0.3255 (2)	0.1708 (4)	0.0543 (13)	
H13	0.598512	0.349448	0.239900	0.065*	
C14	0.5465 (6)	0.2439 (3)	−0.0011 (5)	0.0778 (18)	
H14A	0.447518	0.256600	−0.014784	0.117*	
H14B	0.553480	0.197671	0.019415	0.117*	
H14C	0.590723	0.252728	−0.081424	0.117*	
C15	0.7682 (6)	0.2678 (3)	0.1477 (6)	0.0799 (18)	
H15A	0.803769	0.296598	0.220213	0.120*	
H15B	0.820744	0.275203	0.071894	0.120*	
H15C	0.779881	0.222732	0.176556	0.120*	

### Dichlorido[2-(3-cyclopentyl-1,2,4-triazol-5-yl-κN4)pyridine-κN]palladium(II) dimethylformamide monosolvate Atomic displacement parameters (Å^2^)

**Table:**

	*U*^11^	*U*^22^	*U*^33^	*U*^12^	*U*^13^	*U*^23^
Pd1	0.02677 (15)	0.02755 (16)	0.03033 (17)	−0.00105 (13)	0.00115 (11)	−0.00292 (13)
Cl1	0.0606 (7)	0.0298 (5)	0.0473 (6)	−0.0059 (5)	0.0080 (5)	−0.0003 (5)
Cl2	0.0589 (7)	0.0423 (6)	0.0431 (6)	−0.0010 (5)	0.0144 (5)	−0.0117 (5)
N1	0.0259 (16)	0.0341 (18)	0.0317 (18)	0.0024 (14)	−0.0002 (13)	−0.0026 (14)
N2	0.0241 (15)	0.0229 (15)	0.0306 (17)	0.0004 (13)	−0.0003 (13)	−0.0014 (13)
N3	0.049 (2)	0.0303 (19)	0.042 (2)	0.0047 (16)	0.0135 (17)	−0.0008 (16)
N4	0.046 (2)	0.037 (2)	0.0342 (19)	0.0060 (16)	0.0126 (16)	−0.0037 (15)
C1	0.056 (3)	0.048 (3)	0.033 (2)	0.005 (2)	0.013 (2)	−0.001 (2)
C2	0.077 (4)	0.059 (3)	0.046 (3)	0.007 (3)	0.020 (3)	0.019 (2)
C3	0.075 (4)	0.039 (3)	0.063 (3)	0.007 (3)	0.018 (3)	0.022 (2)
C4	0.057 (3)	0.033 (2)	0.052 (3)	0.006 (2)	0.012 (2)	0.007 (2)
C5	0.028 (2)	0.033 (2)	0.037 (2)	0.0005 (17)	−0.0003 (17)	0.0011 (18)
C6	0.030 (2)	0.028 (2)	0.035 (2)	−0.0012 (17)	0.0003 (17)	−0.0010 (17)
C7	0.0252 (19)	0.037 (2)	0.032 (2)	0.0006 (17)	0.0019 (16)	0.0001 (17)
C8	0.0318 (19)	0.040 (2)	0.031 (2)	−0.0023 (18)	0.0002 (16)	−0.0001 (18)
C9	0.055 (3)	0.062 (3)	0.033 (2)	−0.005 (2)	0.002 (2)	0.005 (2)
C10	0.077 (3)	0.098 (4)	0.056 (3)	−0.025 (3)	0.008 (3)	0.024 (3)
C11	0.080 (4)	0.124 (5)	0.080 (4)	−0.046 (3)	−0.009 (3)	0.043 (3)
C12	0.051 (3)	0.072 (3)	0.056 (3)	−0.024 (3)	−0.009 (2)	0.016 (3)
O1	0.085 (3)	0.071 (3)	0.056 (2)	0.029 (2)	0.026 (2)	−0.0065 (18)
N5	0.054 (2)	0.034 (2)	0.055 (2)	−0.0022 (18)	0.0126 (19)	−0.0088 (17)
C13	0.087 (4)	0.043 (3)	0.036 (3)	0.009 (3)	0.019 (3)	−0.002 (2)
C14	0.066 (4)	0.071 (4)	0.095 (5)	0.008 (3)	0.000 (3)	−0.038 (3)
C15	0.066 (4)	0.059 (4)	0.115 (5)	−0.006 (3)	0.012 (4)	−0.028 (3)

### Dichlorido[2-(3-cyclopentyl-1,2,4-triazol-5-yl-κN4)pyridine-κN]palladium(II) dimethylformamide monosolvate Geometric parameters (Å, º)

**Table:**

Pd1—Cl1	2.2811 (11)	C8—C12	1.528 (6)
Pd1—Cl2	2.2837 (10)	C9—H9A	0.9700
Pd1—N1	2.061 (3)	C9—H9B	0.9700
Pd1—N2	2.038 (3)	C9—C10	1.511 (6)
N1—C1	1.348 (5)	C10—H10A	0.9700
N1—C5	1.352 (5)	C10—H10B	0.9700
N2—C6	1.374 (5)	C10—C11	1.487 (7)
N2—C7	1.334 (4)	C11—H11A	0.9700
N3—N4	1.358 (4)	C11—H11B	0.9700
N3—C6	1.312 (5)	C11—C12	1.514 (7)
N4—H4	0.8600	C12—H12A	0.9700
N4—C7	1.328 (5)	C12—H12B	0.9700
C1—H1	0.9300	O1—C13	1.225 (6)
C1—C2	1.376 (6)	N5—C13	1.308 (5)
C2—H2	0.9300	N5—C14	1.440 (6)
C2—C3	1.376 (6)	N5—C15	1.444 (6)
C3—H3	0.9300	C13—H13	0.9300
C3—C4	1.375 (6)	C14—H14A	0.9600
C4—H4A	0.9300	C14—H14B	0.9600
C4—C5	1.373 (5)	C14—H14C	0.9600
C5—C6	1.462 (5)	C15—H15A	0.9600
C7—C8	1.487 (5)	C15—H15B	0.9600
C8—H8	0.9800	C15—H15C	0.9600
C8—C9	1.522 (5)		
			
Cl1—Pd1—Cl2	89.29 (4)	C8—C9—H9A	111.1
N1—Pd1—Cl1	177.27 (9)	C8—C9—H9B	111.1
N1—Pd1—Cl2	93.27 (9)	H9A—C9—H9B	109.0
N2—Pd1—Cl1	96.18 (9)	C10—C9—C8	103.5 (4)
N2—Pd1—Cl2	174.47 (9)	C10—C9—H9A	111.1
N2—Pd1—N1	81.25 (12)	C10—C9—H9B	111.1
C1—N1—Pd1	126.8 (3)	C9—C10—H10A	110.5
C1—N1—C5	118.3 (4)	C9—C10—H10B	110.5
C5—N1—Pd1	115.0 (2)	H10A—C10—H10B	108.7
C6—N2—Pd1	111.2 (2)	C11—C10—C9	106.1 (4)
C7—N2—Pd1	144.6 (3)	C11—C10—H10A	110.5
C7—N2—C6	104.2 (3)	C11—C10—H10B	110.5
C6—N3—N4	102.1 (3)	C10—C11—H11A	110.1
N3—N4—H4	123.9	C10—C11—H11B	110.1
C7—N4—N3	112.2 (3)	C10—C11—C12	108.0 (4)
C7—N4—H4	123.9	H11A—C11—H11B	108.4
N1—C1—H1	119.3	C12—C11—H11A	110.1
N1—C1—C2	121.4 (4)	C12—C11—H11B	110.1
C2—C1—H1	119.3	C8—C12—H12A	110.9
C1—C2—H2	120.1	C8—C12—H12B	110.9
C1—C2—C3	119.8 (4)	C11—C12—C8	104.4 (4)
C3—C2—H2	120.1	C11—C12—H12A	110.9
C2—C3—H3	120.4	C11—C12—H12B	110.9
C4—C3—C2	119.2 (4)	H12A—C12—H12B	108.9
C4—C3—H3	120.4	C13—N5—C14	120.0 (4)
C3—C4—H4A	120.7	C13—N5—C15	122.3 (4)
C5—C4—C3	118.7 (4)	C14—N5—C15	117.7 (4)
C5—C4—H4A	120.7	O1—C13—N5	125.2 (5)
N1—C5—C4	122.6 (4)	O1—C13—H13	117.4
N1—C5—C6	113.0 (3)	N5—C13—H13	117.4
C4—C5—C6	124.4 (4)	N5—C14—H14A	109.5
N2—C6—C5	119.6 (3)	N5—C14—H14B	109.5
N3—C6—N2	113.7 (3)	N5—C14—H14C	109.5
N3—C6—C5	126.6 (4)	H14A—C14—H14B	109.5
N2—C7—C8	127.7 (4)	H14A—C14—H14C	109.5
N4—C7—N2	107.8 (3)	H14B—C14—H14C	109.5
N4—C7—C8	124.4 (3)	N5—C15—H15A	109.5
C7—C8—H8	108.1	N5—C15—H15B	109.5
C7—C8—C9	116.0 (4)	N5—C15—H15C	109.5
C7—C8—C12	113.2 (3)	H15A—C15—H15B	109.5
C9—C8—H8	108.1	H15A—C15—H15C	109.5
C9—C8—C12	103.0 (3)	H15B—C15—H15C	109.5
C12—C8—H8	108.1		
			
Pd1—N1—C1—C2	−178.4 (3)	C2—C3—C4—C5	−0.8 (7)
Pd1—N1—C5—C4	177.8 (3)	C3—C4—C5—N1	1.2 (7)
Pd1—N1—C5—C6	−0.1 (4)	C3—C4—C5—C6	178.8 (4)
Pd1—N2—C6—N3	−178.2 (3)	C4—C5—C6—N2	−177.5 (4)
Pd1—N2—C6—C5	−0.5 (4)	C4—C5—C6—N3	0.0 (6)
Pd1—N2—C7—N4	177.4 (3)	C5—N1—C1—C2	−0.1 (6)
Pd1—N2—C7—C8	−1.0 (7)	C6—N2—C7—N4	−0.1 (4)
N1—C1—C2—C3	0.4 (8)	C6—N2—C7—C8	−178.5 (3)
N1—C5—C6—N2	0.4 (5)	C6—N3—N4—C7	0.1 (4)
N1—C5—C6—N3	177.8 (4)	C7—N2—C6—N3	0.2 (4)
N2—C7—C8—C9	−154.3 (4)	C7—N2—C6—C5	178.0 (3)
N2—C7—C8—C12	86.9 (5)	C7—C8—C9—C10	−163.2 (4)
N3—N4—C7—N2	0.0 (4)	C7—C8—C12—C11	158.7 (4)
N3—N4—C7—C8	178.4 (3)	C8—C9—C10—C11	30.6 (6)
N4—N3—C6—N2	−0.2 (4)	C9—C8—C12—C11	32.7 (5)
N4—N3—C6—C5	−177.7 (4)	C9—C10—C11—C12	−10.2 (7)
N4—C7—C8—C9	27.7 (5)	C10—C11—C12—C8	−14.2 (7)
N4—C7—C8—C12	−91.1 (5)	C12—C8—C9—C10	−39.0 (5)
C1—N1—C5—C4	−0.7 (6)	C14—N5—C13—O1	1.9 (8)
C1—N1—C5—C6	−178.6 (3)	C15—N5—C13—O1	−178.4 (5)
C1—C2—C3—C4	0.0 (8)		

### Dichlorido[2-(3-cyclopentyl-1,2,4-triazol-5-yl-κN4)pyridine-κN]palladium(II) dimethylformamide monosolvate Hydrogen-bond geometry (Å, º)

**Table:**

*D*—H···*A*	*D*—H	H···*A*	*D*···*A*	*D*—H···*A*
N4—H4···O1	0.86	1.85	2.679 (4)	161
C3—H3···N3^i^	0.93	2.66	3.471 (6)	146

Symmetry code: (i) *x*, −*y*+1/2, *z*+1/2.

## References

[bb1] Abu-Surrah, A. & Kettunen, M. (2006). *Curr. Med. Chem.* **13**, 1337–1357. 10.2174/092986706776872970 16712474

[bb2] Bruker (2014). *APEX2* and *SAINT*. Bruker AXS Inc., Madison, Wisconsin, USA.

[bb3] Cuerva, C., Campo, J. A., Cano, M. & Lodeiro, C. (2019). *Chem. Eur. J.* **25**, 12046–12051. 10.1002/chem.201901763 31237959

[bb4] Cuerva, C., Campo, J. A., Cano, M. & Schmidt, R. (2019). *J. Mater. Chem. C.* **7**, 10318–10330.

[bb5] Cuerva, C., Campo, J. A., Cano, M., Schmidt, R. & Lodeiro, C. (2018). *J. Mater. Chem. C.* **6**, 9723–9733.

[bb6] Cuerva, C., Campo, J. A., Ovejero, P., Torres, M. R. & Cano, M. (2014). *Dalton Trans.* **43**, 8849–8860. 10.1039/c4dt00369a 24781448

[bb7] Cuerva, C., Cano, M. & Schmidt, R. (2023). *Dalton Trans.* **52**, 4684–4691. 10.1039/d2dt03754h 36779291

[bb8] Dolomanov, O. V., Bourhis, L. J., Gildea, R. J., Howard, J. A. K. & Puschmann, H. (2009). *J. Appl. Cryst.* **42**, 339–341.

[bb9] Ervithayasuporn, V. (2016). *CSD Communication* (CCDC 1053096). CCDC, Cambridge, England.

[bb10] Ervithayasuporn, V., Kwanplod, K., Boonmak, J., Youngme, S. & Sangtrirutnugul, P. (2015). *J. Catal.* **332**, 62–69.

[bb32] Groom, C. R., Bruno, I. J., Lightfoot, M. P. & Ward, S. C. (2016). *Acta Cryst*. B**72**, 171–179. 10.1107/S2052520616003954 PMC4822653 27048719

[bb11] Ivanova, H. V., Khomenko, D. M., Doroshchuk, R. O., Stoica, A.-C., Zakharchenko, B. V., Rusanova, J. A., Raspertova, I. V., Shova, S. & Lampeka, R. D. (2024). *ChemistrySelect*, https://doi. org/10.1002/slct. 202402258.

[bb12] Jakubowski, M., Łakomska, I., Sitkowski, J., Pokrywczyńska, M., Dąbrowski, P., Framski, G. & Ostrowski, T. (2020). *Polyhedron*, **180**, 114428.

[bb13] Jiao, L.-Y., Yin, X.-M., Liu, S., Zhang, Z., Sun, M. & Ma, X.-X. (2020). *Catal. Commun.* **135**, 105889.

[bb14] Jindabot, S., Teerachanan, K., Thongkam, P., Kiatisevi, S., Khamnaen, T., Phiriyawirut, P., Charoenchaidet, S., Sooksimuang, T., Kongsaeree, P. & Sangtrirutnugul, P. (2014). *J. Organomet. Chem.* **750**, 35–40.

[bb16] Khomenko, D. N., Doroschuk, R. A. & Lampeka, R. D. (2009). *Ukr. J. Chem.* **75**, 30–33.

[bb17] Krause, L., Herbst-Irmer, R., Sheldrick, G. M. & Stalke, D. (2015). *J. Appl. Cryst.* **48**, 3–10. 10.1107/S1600576714022985 PMC4453166 26089746

[bb18] Kumbhar, A. (2017). *J. Organomet. Chem.* **848**, 22–88.

[bb19] Lang, C., Kiefer, C., Lejeune, E., Goldmann, A. S., Breher, F., Roesky, P. W. & Barner-Kowollik, C. (2012). *Polym. Chem.* **3**, 2413–2420.

[bb20] McKinnon, J. J., Jayatilaka, D. & Spackman, M. A. (2007). *Chem. Commun.* pp. 3814–3816. 10.1039/b704980c 18217656

[bb21] Ohorodnik, Y. M., Khomenko, D. M., Doroshchuk, R. O., Raspertova, I. V., Shova, S., Babak, M. V., Milunovic, M. N. M. & Lampeka, R. D. (2023). *Inorg. Chim. Acta*, **556**, 121646.

[bb22] Ouellette, V., Côté, M.-F., Gaudreault, R. C., Tajmir-Riahi, H.-A. & Bérubé, G. (2019). *Eur. J. Med. Chem.* **179**, 660–666. 10.1016/j.ejmech.2019.06.090 31279298

[bb23] Schweinfurth, D., Pattacini, R., Strobel, S. & Sarkar, B. (2009). *Dalton Trans.* pp. 9291–9297. 10.1039/b910660j 20449208

[bb24] Schweinfurth, D., Strobel, S. & Sarkar, B. (2011). *Inorg. Chim. Acta*, **374**, 253–260.

[bb25] Sheldrick, G. M. (2015*a*). *Acta Cryst.* A**71**, 3–8.

[bb26] Sheldrick, G. M. (2015*b*). *Acta Cryst.* C**71**, 3–8.

[bb27] Spackman, M. A. & Jayatilaka, D. (2009). *CrystEngComm*, **11**, 19–32.

[bb28] Spackman, P. R., Turner, M. J., McKinnon, J. J., Wolff, S. K., Grimwood, D. J., Jayatilaka, D. & Spackman, M. A. (2021). *J. Appl. Cryst.* **54**, 1006–1011. 10.1107/S1600576721002910 PMC8202033 34188619

[bb29] Yano, S., Ohi, H., Ashizaki, M., Obata, M., Mikata, Y., Tanaka, R., Nishioka, T., Kinoshita, I., Sugai, Y., Okura, I., Ogura, S., Czaplewska, J. A., Gottschaldt, M., Schubert, U. S., Funabiki, T., Morimoto, K. & Nakai, M. (2012). *Chem. Biodivers.* **9**, 1903–1915. 10.1002/cbdv.201100426 22976979

[bb30] Zakharchenko, B. V., Khomenko, D. M., Doroschuk, R. O., Raspertova, I. V., Shova, S., Grebinyk, A. G., Grynyuk, I. I., Prylutska, S. V., Matyshevska, O. P., Slobodyanik, M. S., Frohme, M. & Lampeka, R. D. (2021). *Chem. Pap.* **75**, 4899–4906.

[bb31] Zakharchenko, B. V., Khomenko, D. M., Doroshchuk, R. O., Raspertova, I. V., Starova, V. S., Trachevsky, V. V., Shova, S., Severynovska, O. V., Martins, L. M. D. R. S., Pombeiro, A. J. L., Arion, V. B. & Lampeka, R. D. (2019). *New J. Chem.* **43**, 10973–10984.

